# SARS-CoV-2 mutations in MHC-I-restricted epitopes evade CD8^+^ T cell responses

**DOI:** 10.1126/sciimmunol.abg6461

**Published:** 2021-03-04

**Authors:** Benedikt Agerer, Maximilian Koblischke, Venugopal Gudipati, Luis Fernando Montaño-Gutierrez, Mark Smyth, Alexandra Popa, Jakob-Wendelin Genger, Lukas Endler, David M. Florian, Vanessa Mühlgrabner, Marianne Graninger, Stephan W. Aberle, Anna-Maria Husa, Lisa Ellen Shaw, Alexander Lercher, Pia Gattinger, Ricard Torralba-Gombau, Doris Trapin, Thomas Penz, Daniele Barreca, Ingrid Fae, Sabine Wenda, Marianna Traugott, Gernot Walder, Winfried F. Pickl, Volker Thiel, Franz Allerberger, Hannes Stockinger, Elisabeth Puchhammer-Stöckl, Wolfgang Weninger, Gottfried Fischer, Wolfgang Hoepler, Erich Pawelka, Alexander Zoufaly, Rudolf Valenta, Christoph Bock, Wolfgang Paster, René Geyeregger, Matthias Farlik, Florian Halbritter, Johannes B. Huppa, Judith H. Aberle, Andreas Bergthaler

**Affiliations:** 1 CeMM Research Center for Molecular Medicine of the Austrian Academy of Sciences, Vienna, Austria.; 2 Center for Virology, Medical University of Vienna, Vienna, Austria.; 3 Institute for Hygiene and Applied Immunology, Center for Pathophysiology, Infectiology and Immunology, Medical University of Vienna, Vienna, Austria.; 4 St. Anna Children´s Cancer Research Institute (CCRI), Vienna, Austria.; 5 Department of Dermatology, Medical University of Vienna, Vienna, Austria.; 6 Department of Pathophysiology and Allergy Research, Division of Immunopathology, Medical University of Vienna, Vienna, Austria.; 7 Institute of Immunology, Center for Pathophysiology, Infectiology and Immunology, Medical University of Vienna, Vienna, Austria.; 8 Department of Blood Group Serology and Transfusion Medicine, Medical University of Vienna, Vienna, Austria.; 9 Department of Medicine 4, Clinic Favoriten, Vienna, Austria.; 10 Division of Hygiene and Medical Microbiology, Medical University of Innsbruck, Innsbruck, Austria.; 11 Karl Landsteiner University of Health Sciences, Krems, Austria.; 12 Institute of Virology and Immunology, Bern and Mittelhäusern, Switzerland.; 13 Department of Infectious Diseases and Pathobiology, Vetsuisse Faculty, University of Bern, Bern, Switzerland.; 14 Austrian Agency for Health and Food Safety (AGES), Vienna, Austria.; 15 Laboratory for Immunopathology, Department of Clinical Immunology and Allergy, First Moscow State Medical University Sechenov, Moscow, Russia.; 16 NRC Institute of Immunology FMBA of Russia, Moscow, Russia.; 17 Department of Laboratory Medicine, Medical University of Vienna, Vienna, Austria.

## Abstract

CD8^+^ T cell immunity to SARS-CoV-2 has been implicated in COVID-19 severity and virus control. Here, we identified nonsynonymous mutations in MHC-I-restricted CD8^+^ T cell epitopes after deep sequencing of 747 SARS-CoV-2 virus isolates. Mutant peptides exhibited diminished or abrogated MHC-I binding in a cell-free in vitro assay. Reduced MHC-I binding of mutant peptides was associated with decreased proliferation, IFN-γ production and cytotoxic activity of CD8^+^ T cells isolated from HLA-matched COVID-19 patients. Single cell RNA sequencing of ex vivo expanded, tetramer-sorted CD8^+^ T cells from COVID-19 patients further revealed qualitative differences in the transcriptional response to mutant peptides. Our findings highlight the capacity of SARS-CoV-2 to subvert CD8^+^ T cell surveillance through point mutations in MHC-I-restricted viral epitopes.

## Teaser

SARS-CoV-2 mutations in MHC-I epitopes identified by deep viral sequencing evade CTL responses through decreased peptide-MHC-I binding.

## INTRODUCTION

SARS-CoV-2 infection elicits broad activation of the innate and adaptive arms of immunity (*1*–*4*). Major correlates of protection are neutralizing antibodies and cytotoxic CD8^+^ T-lymphocytes (CTLs) (*5*). CTLs play an essential role in conferring immune memory and protection against viral pathogens (*6*–*8*). CTLs kill infected cells upon recognition of viral epitopes as they are displayed on the cell surface in the context of the class I major histocompatibility complex proteins (MHC-I). Certain positions in these epitopes have been shown to be critical for MHC-I presentation and mutations in these so-called anchor residues might interfere with peptide binding to MHC-I (*9*, *10*). CTL responses have been described in great detail in SARS-CoV-2–infected patients (*3*, *4*, *11*–*15*). In acute SARS-CoV-2 infection, CTLs show high levels of cytotoxic effector molecules such as granzyme B, perforin and IFN-γ (*16*). Numerous human leukocyte antigen (HLA)-restricted CTL epitopes have been characterized for SARS-CoV-2 (*4*, *17*–*22*).

Compelling evolutionary evidence for CTL-mediated control of RNA viruses causing chronic infections like HIV and HCV is provided by mutations occurring in viral epitopes which directly interfere with MHC-I-restricted T cell antigen recognition and killing by CTLs (*24*–*27*). While several mutations in SARS-CoV-2 have recently been associated with an escape from antibody responses (*28*–*30*), the extent to which SARS-CoV-2 mutations may upend the presentation of virus-derived peptides via MHC-I remains to be determined. In this study, we used deep viral genome sequencing to identify nonsynonymous mutations in previously reported MHC-I epitopes. We applied a combination of cell-free in vitro assays, as well as functional and transcriptional characterization of COVID-19 patient-derived PBMCs to investigate the potential of single point mutations in MHC-I epitopes to evade CTL responses.

## RESULTS

### Bioinformatic analysis of mutations in putative SARS-CoV-2 CD8^+^ T cell epitopes

**Fig. 1:**
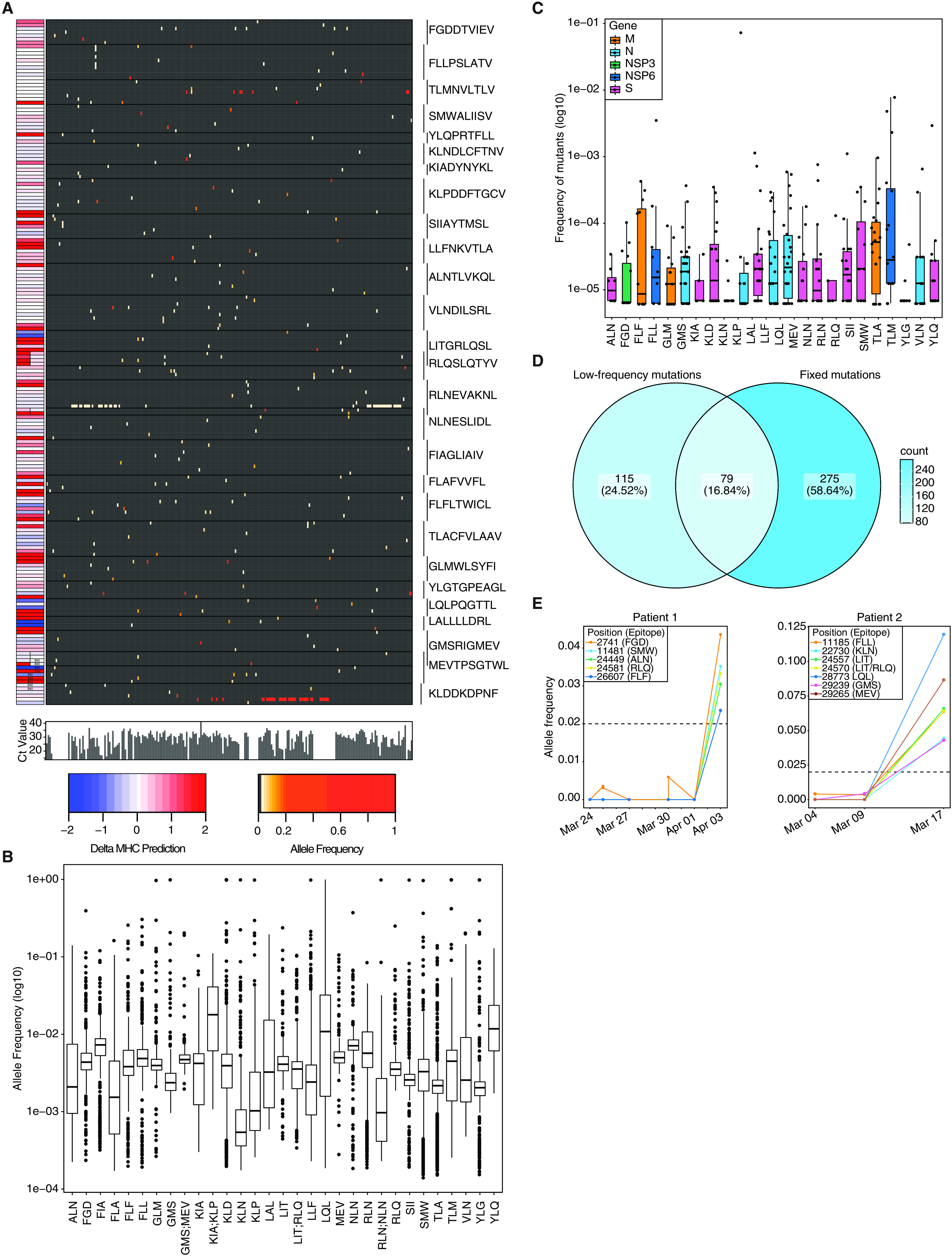
**Nonsynonymous mutations are detected in SARS-CoV-2 CTL epitopes. A)** Allele frequency of low-frequency mutations detected in 27 CTL epitopes. Epitopes are indicated on the right. The heatmap to the left indicates change in % ranks predicted by netMHCpan 4.1 (*32*). Bar plots below the large heatmap indicate viral loads as Ct values. **B)** Allele frequency of mutations in specified epitopes. Regions present in two epitopes are depicted separately. **C)** Frequency of global fixed mutations in CTL epitopes. **D)** Venn diagram depicting overlap between global fixed mutations and low-frequency variants. **E)** Mutations in CTL epitopes arise late in infection. Mutation frequency over time of two patients which were longitudinally sampled. Shown are variants that lead to nonsynonymous mutations in CTL epitopes. Patient 1 was sampled multiple times on the same day for some time points. Dashed lines indicate the detection limit for calling low-frequency mutations.

To assess a possible impact of virus mutations on SARS-CoV-2-specific CD8^+^ T cell responses, we performed deep viral genome sequencing (> 20,000X coverage) (Fig. S1A) and bioinformatic analysis on 747 SARS-CoV-2 samples (21). We focused on 27 CTL epitopes, which had previously been reported as experimentally-validated epitopes restricted by HLA-A*02:01 (allele frequency 0.29 in Austria) or HLA-B*40:01 (allele frequency 0.03-0.05 in Austria) (*4*, *17*–*20*, *31*). Most of the selected epitopes are located in the S protein (N=13) and the remaining epitopes are distributed between the N (N=6), ORF1ab (N=4), M (N=3) and E (N=1) proteins. Detailed descriptions of peptides and their three-letter code are listed in Table S1. Among these epitopes we detected 194 nonsynonymous mutations present at frequencies of ≥ 0.02 in 229 samples (Fig. 1A-B). Of these 194 variants, 35 were found at frequencies between 0.1 to 0.5. Notably, 9 variants were fixed (frequency ≥ 0.9) and found in 53 different patient samples (Table S2). Due to overlaps in some epitopes, these 194 mutations result in 199 different epitope variants. Forty-one of these mutations were localized to anchor residues, and 21 mutations affected auxiliary residues, which are both integral to MHC-I peptide loading (Fig. S1B) (*9*, *10*). Prediction of the binding strength of the wild type and mutant peptides to HLA-A*02:01 and HLA-B*40:01 via NetMHCpan v4.1 (*32*) revealed weaker peptide binding to MHC-I, as indicated by an increase of NetMHCpan % ranks (Fig. S1C-F). For many of the investigated CTL epitopes, we detected multiple variants that independently emerged in different SARS-CoV-2 infected individuals (Fig. 1A). To corroborate these findings from low-frequency mutations in our deep sequencing dataset, we analyzed fixed mutations in >145,000 available global SARS-CoV-2 sequences from the public database GISAID (*33*). Mutations were observed in 0.0000689 - 7.336% epitope sequences (mean = 0.005106%) (Fig. 1C). We found 10 to 11,717 viral genome sequences with a nonsynonymous mutation for each of the investigated 27 CTL epitopes (mean = 807.05). Importantly, we found fixed variants in GISAID that were also identified in our low-frequency analysis, highlighting the relevance of individual low-frequency mutations (Fig. 1D, S1G-S1H).

To examine the time dynamics of low-frequency epitope mutations in patients, we utilized serially-sampled viral genomes from COVID-19 patients. Suggestive of CTL-mediated selection pressures, mutations in viral epitopes arose typically later in infection (Fig. 1E). Based on our analysis of low-frequency and fixed mutations, we selected 11 wild type and 17 corresponding mutant peptides with predicted decreased HLA-binding strength for further biophysical and functional analyses (Table S3).

### Mutations in CTL epitopes destabilize MHC-I complexes

**Fig. 2:**
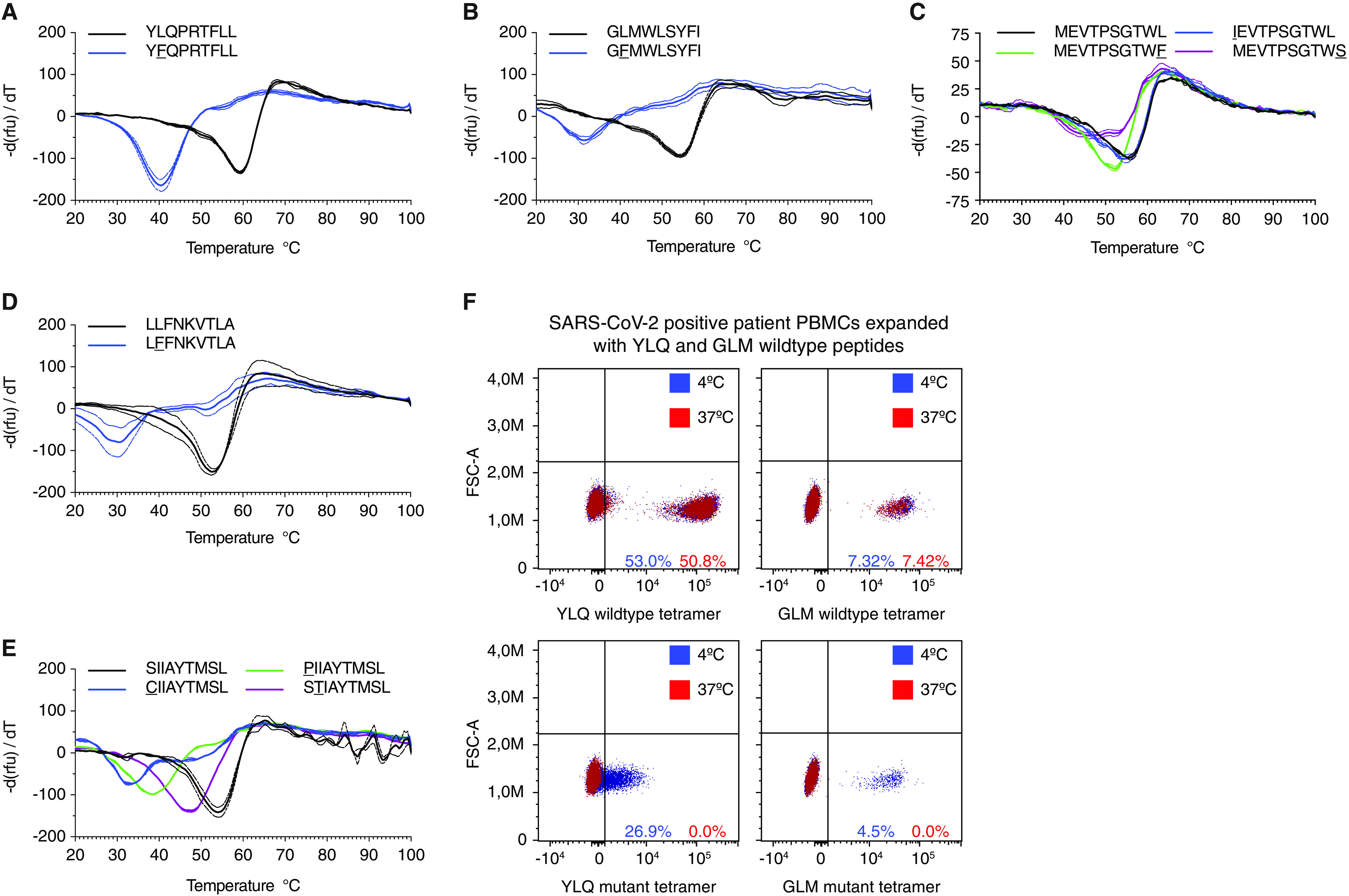
**Epitope variants lead to diminished MHC-I binding. A-E**) Decreased thermostability of mutant peptide MHC-I complexes. Negative first derivative of relative fluorescence units (rfu) plotted against increasing temperatures. Curves for wild type peptides are black, mutated peptides are colored. The minimum point of the curves represents the melting temperature of peptide-MHC-I complexes. Dashed lines indicate SD. n=2-3 technical replicates. **F)** Tetramers featuring mutated peptides are unstable at 37°C. FACS plots showing staining of in vitro expanded PBMCs stained with tetramers containing wild type (top) or mutant (bottom) peptides incubated at 4°C (blue) or 37°C (red).

To assess the MHC-I dependent immunogenic properties of nonsynonymous mutant peptides we produced MHC-I complexes with ultraviolet light cleavable peptides (UVCP) and performed peptide exchange reactions to destabilize and later reassemble MHC-I UVCP complexes (*34*). We next employed cell-free differential scanning fluorimetry (DSF) to measure the thermal stability of destabilized or reassembled MHC-I complexes (Fig. 2A-E, S2A-L) (*35*–*37*). As shown in Fig. S1A and S1B, HLA-A*02:01-UVCP complex is destabilized upon exposure to UV light and can be reassembled by adding UVCP peptide after UV exposure. The minima of the curves specify the melting temperature (T_m_) of the HLA-peptide complexes. T_m_ values well above 37°C indicate strong peptide binding to MHC-I at physiological temperatures, whereas values around 37°C correlate only with weak and below 36°C with absent binding. For 9 of the 11 wild type peptides we observed binding to HLA-A*02:01 or HLA-B*40:01, indicating that these peptides can in principle be presented by the respective HLA allele (Fig. S2C and S2D). In contrast, 11 analyzed mutants exhibited decreased stabilizing capacity toward MHC-I (Fig. 2A-E, S2D, S2F-S2L, Table S4). The HLA-B*40:01–restricted MEVTPSGTWL peptide featured specific binding to recombinant HLA-B*40:01 but not to HLA-A*02:01 (*4*) (Fig. 2C, S2L). An example of a weak binder is the mutant variant YFQPRTFLL (instead of YLQPRTFLL), whereas LFFNKVTLA (instead of LLFNKVTLA) represents a non-binder for HLA-A*02:01 (Fig. 2A, D). Of note, we did neither observe binding of the wild type nor the mutant peptides to HLA-A*02:01 for the published CTL epitopes LQLPQGTTL and LALLLLDRL (Fig. S2I and S2L).

To further corroborate these results, we generated peptide-loaded HLA-A*02:01 and HLA-B*40:01 tetramers presenting wild type and mutant peptides as a means to identify cognate CD8^+^ T cells from expanded PBMCs of HLA-matched COVID-19 patients. As shown in Fig. 2F, tetramers loaded with mutant peptides stain cognate T cells in a T-cell receptor (TCR)-dependent fashion when kept at 4°C. However, when tetramers were incubated at 37°C prior to their use, T cell staining was abrogated, most likely due to peptide loss and structural disintegration of MHC-I. Taken together, these results imply that mutations found in SARS-CoV-2 genome sequences decrease peptide-MHC-I stability and subsequently could promote immune evasion from HLA-dependent recognition by CTLs.

### Investigation of epitope responses in SARS-CoV-2 positive patient PBMCs

**Fig. 3:**
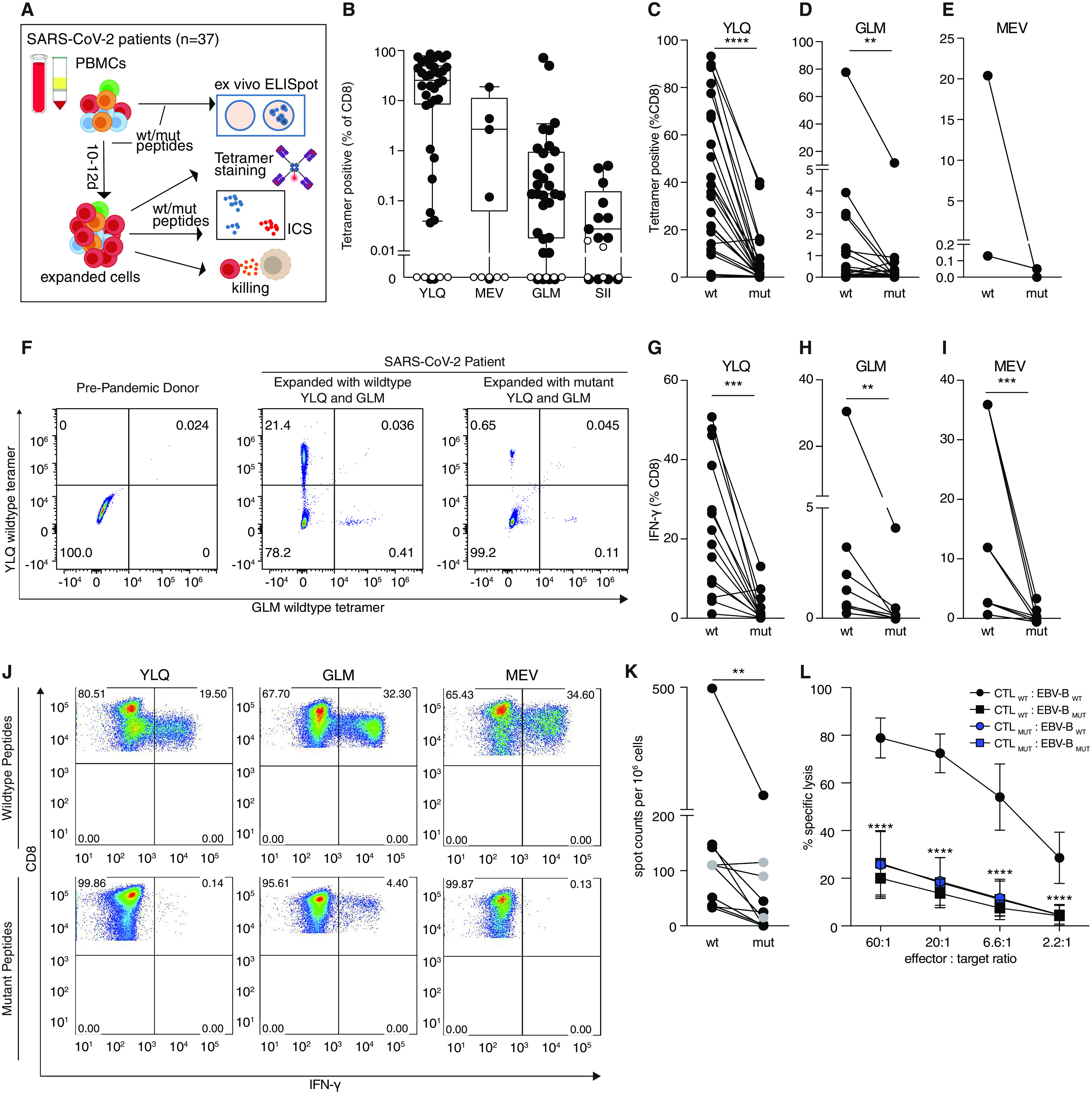
**SARS-CoV-2 epitope mutations are associated with decreased CTL responses. A)** Experimental overview. **B)** CTL responses against wild type epitopes. PBMCs were isolated from HLA-A*02:01 or HLA-B*40:01 positive SARS-CoV-2 patients (black, n=35, 5, 3, or 13 respectively, or pre-pandemic controls with unknown HLA status (white, n=7), expanded 10-12 days with indicated peptides, and stained with wild type tetramers. Boxes show median ± 25^th^ and 75^th^ percentile and whiskers indicate 10^th^ and 90^th^ percentile. **C-E)** T cells expanded with mutant peptides do not give rise to wild type peptide-specific CTLs. PBMCs were isolated as in B), stimulated with wild type or mutant peptides and stained with tetramers containing the wild type peptide. (n=27, 25, and 2 patients per epitope). **F)** Representative FACS plots for C-E. **G-I)** Impact of mutations on CTL response. PBMCs expanded with wild type or mutant peptides as indicated, were analyzed for IFN-γ-production via ICS after restimulation with wild type or mutant peptide (n=14, 8, and 4 patients per epitope). **J)** Representative FACS plots for G-I. **K)** Ex vivo IFN-γ ELISpot assays from PBMCs stimulated with the YLQ peptide or the corresponding mutant (n=7, PBMCs obtained 2.7 ± 0.8 weeks after symptom onset) or the MEV peptide (marked in gray) or corresponding mutant (n=1, PBMCs obtained 3 weeks after symptom onset). Two or three wells were evaluated per sample and peptide. Patient ID is as indicated in Table S6. **L)** CTL killing assay. PBMCs from 4 patients were expanded with wild type or mutant YLQ peptide, mixed with autologous EBV^+^ B cells that were pulsed with wild type or mutant YLQ peptide and specific killing was assessed (n=2 per patient). Error bars represent mean ± SD. Significance is indicated as *P<0.05, **P<0.01, ***P<0.001, ****P<0.0001, tested by Wilcoxon matched-pairs signed rank test (C,D,E,G,H,I,K) or 2-way ANOVA followed by Dunnett’s multiple comparison test (L).

Next, we investigated peptide-specific CD8^+^ T cell responses in PBMCs isolated from HLA-A*02:01 or HLA-B*40:01 positive COVID-19 patients (Fig. 3A, Table S5, Table S6). We first screened the 9 wild type peptides, which showed MHC-I binding in the DSF assay, using ex vivo ELISpot assays (Table S7). Of note, none of the 5 pre-pandemic healthy controls gave responses to any of the peptides. Further, we could detect a positive response in at least one patient for four of the peptides, which were investigated in additional assays. To this end HLA-matched PBMCs from COVID-19 patients and 5 pre-pandemic controls were stimulated with peptides and cultured for 10-12 days followed by tetramer staining (Fig. 3B). This allowed us to confirm these wild type peptides as bona fide T cell epitopes in SARS-CoV-2. We further corroborated virus-specific CTL responses by intracellular cytokine staining (ICS) for IFN-γ after peptide-mediated restimulation (Fig. S3A, B). To investigate the extent to which identified mutations in viral epitopes affected T cell proliferation, we stained wild type or mutant peptide expanded PBMCs with wild type peptide-loaded HLA tetramers. We found fewer tetramer-positive CD8^+^ T cells in PBMCs expanded in the presence of mutant peptides, indicating that mutant peptides featured significantly reduced immunogenicity (Fig. 3C-F). Consistent with this, ICS for IFN-γ revealed significantly diminished CTL responses after restimulation with mutant peptide as compared to the corresponding wild type peptides (Fig. 3G-J). We observed markedly diminished CTL responses to several mutant peptides both presented in the context of HLA-A*02:01 and HLA-B*40:01. This was further supported by peptide titration experiments involving wild type peptides and their mutant counterparts for T cell stimulation (Fig. S3C). To complement intracellular cytokine measurements, we also carried out ex vivo ELISpot assays after stimulating PBMCs with wild type or mutant peptides without extended expansion. These experiments confirmed that stimulation with mutant peptides resulted in significantly fewer cytokine-producing cells than with wild type peptides (Fig. 3K, S3D). To further corroborate these results, we performed functional cytotoxicity assays with PBMCs isolated from four COVID-19 patients expanded for 10-12 days in the presence of wild type or mutant YLQPRTFLL (YLQ) peptide. We assessed the ability of these cells to kill autologous Epstein-Barr virus (EBV)-transformed lymphoblastoid B cell lines (EBV^+^ B cells) that were pulsed with either wild type or mutant YLQ. While wild type expanded PBMCs showed specific killing of wild type pulsed EBV^+^ cells, they failed to kill EBV^+^ B cells pulsed with mutant peptide (Fig. 3L), suggesting that mutant YLQ is not properly presented by the EBV^+^ B cells. Furthermore, PBMCs expanded with mutant peptides failed to kill both wild type and mutant peptide pulsed EBV^+^ B cells (Fig. 3L and S3E). These results further underline that expansion with the mutant peptide failed to mount a functional CTL response.

### Transcriptional single T cell analysis upon stimulation with wild type or mutant peptides

**Fig. 4:**
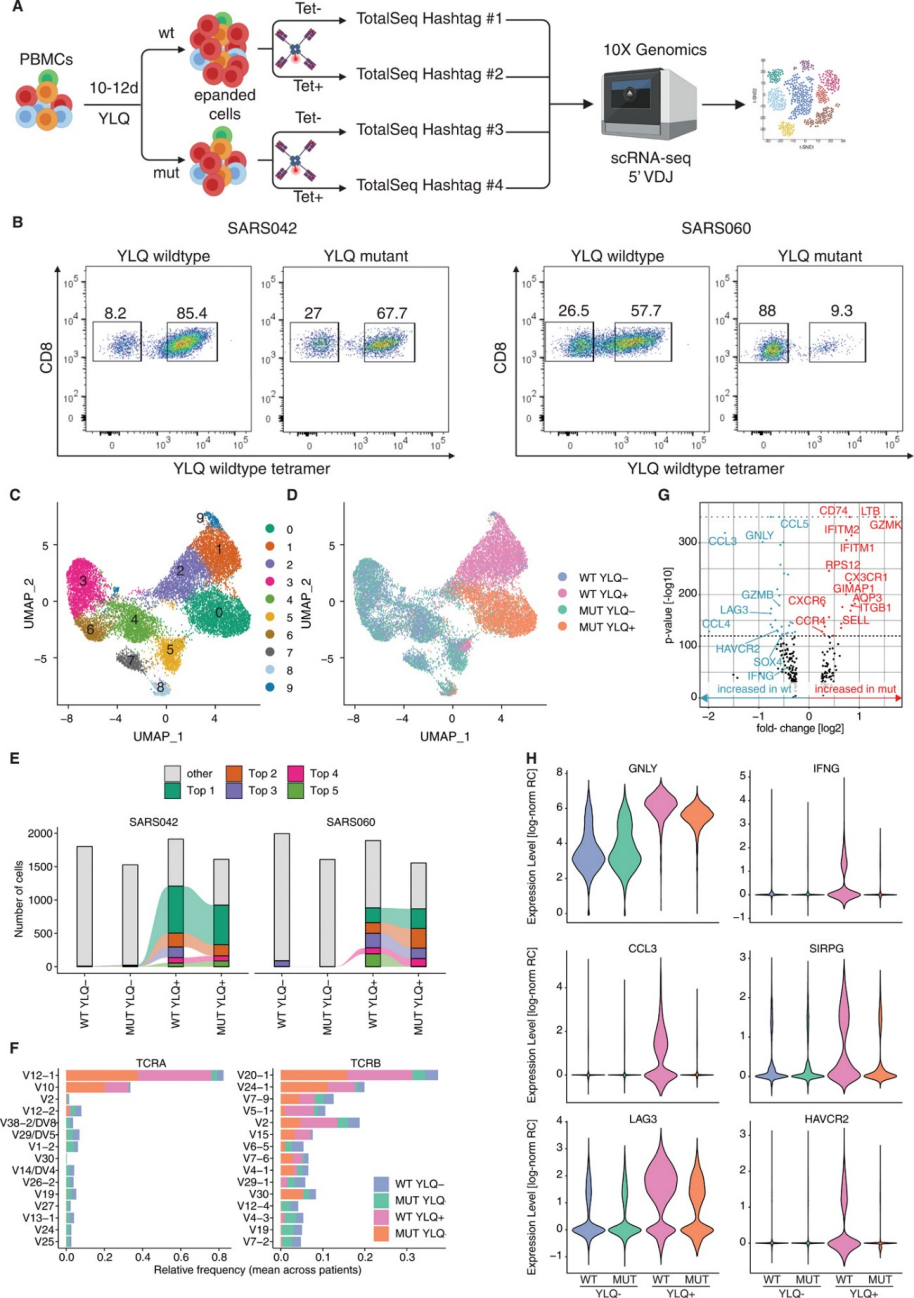
**Single cell transcriptomics and TCR sequencing of CD8^+^ T cells reveals distinct transcritptional profiles in response to mutant peptide. A)** Experimental setup. PBMCs were expanded for 10-12 days in the presence of wild type or mutant YLQ peptide, sorted for YLQ tetramer-positive and tetramer-negative CD8^+^ cells, labeled with barcoded antibodies (TotalSeq anti-human Hashtag) and subjected to single-cell RNA sequencing (figure generated with BioRender.com). **B)** Percentages of YLQ tetramer-positive CD8^+^ T cells in response to wild type or mutant peptide expansion from the two donors analyzed. **C-D)** UMAP plots displaying an embedding of single-cell transcriptomes in 2-dimensional space. The cells are colored according to their clusters (C), or experimental condition (D). **E)** Distribution of clonotypes for both patients and the indicated conditions. The top 5 clonotypes are colored. Connecting lines show clonotypes shared between conditions. **F)** Top 15 TRAV and TRVB genes. **G)** Volcano plot displaying differentially expressed genes between wild type-positive and mutant-positive cells. P-values of 0 were capped to 10^−350^ (indicated by gray dotted line). **H)** Violin plots showing expression levels in tetramer-negative and tetramer-positive cells expanded with mutant or wild type peptide. Expression levels given as log-normalized relative read counts (RC). All plots in C-H show combined data from both patients.

To further characterize our results from patient PBMCs we expanded PBMCs isolated from two patients (SARS042 and SARS060) for 10-12 days in the presence of wild type or mutant YLQ peptide. We sorted equal numbers of YLQ tetramer-positive and tetramer-negative CD8^+^ T cells for each condition, labeled them with oligonucleotide-barcoded antibodies (TotalSeq anti-human Hashtag) and performed single-cell RNA sequencing (scRNA-seq) combined with TCR sequencing on a total of 17635 cells (Fig. 4A, S4A). We again noted that expansion elicited by the mutant YLQ peptide resulted in reduced numbers of YLQ tetramer positive cells, consistent with our previous results (Fig. 4B). This unbiased sequencing approach led to the identification of 10 distinct clusters, showing a clear division between tetramer negative and tetramer positive cells (Fig. 4C, S4B). For tetramer positive (responding) cells we identified differential clustering between wild type- and mutant-stimulated cells, indicating that stimulation with mutant peptides not only leads to reduced expansion, but also altered gene expression in tetramer-specific CD8^+^ T cells (Fig. 4D). In contrast, tetramer negative (nonresponding) wild type and mutant-stimulated cells clustered in mixed neighborhoods, further suggesting that differences can only be found in responding cells.

We next analyzed the TCR sequences of these cells. In tetramer negative cells we found a high diversity of TCR sequences for both wild type and mutant peptide conditions (Fig. 4E, S4C). In response to peptide stimulation, we found that the pool of cells consisted of a subset of clones, with 5 T cell clones making up more than 50% of T cells in both patients (Fig. 4E). Importantly, we found the TRAV12-1 gene to be the dominant TRAV variant for both patients, as well as the two TRBV variants TRBV7-9 and TRBV2 to be prominent, which were all recently found to be part of public TCRs specific for YLQ (*19*, *22*) (Fig. 4F). We further asked whether there are T cell clones that specifically expand in response to the mutant peptide. Interestingly, we discovered expansion of the same T cell clones upon stimulation with either wild type or mutant peptide (Fig. 4E).

We next investigated gene expression signatures associated with cytotoxic activity and exhaustion (*38*). Importantly, cytotoxic gene signatures were found enriched in tetramer positive cells (as compared to tetramer negative cells). In line with our cytotoxicity assay results we found up-regulated expression of cytotoxicity associated genes, such as *GZMB*, *PRF1* and *NKG7*, and decreased expression of genes associated with naïve T cell states, such as *IL7R* and *TCF7* (Fig. S4D and S4E). To further our understanding of qualitative differences we performed differential gene expression analysis to compare wild type and mutant peptide-stimulated cells (Fig. 4G). We identified lower expression levels of several cytotoxicity- and exhaustion-associated transcripts such as *GZMB*, *GNLY*, the coinhibitory receptors *LAG3* and *HAVCR2,* the cytokines *IFNG*, *CCL3* and *CCL4* and the costimulatory gene *SIRPG* in mutant-stimulated T cells (Fig. 4G, 4H and S4G). This is in line with a more profound exhaustion gene signature, that has recently been identified in SARS-CoV-2-specific CD8^+^ T cells in cells stimulated with wild type peptide (Fig. S4F). This signature was linked in the literature to a higher expression of cytotoxicity-associated genes (*38*). These findings further underline qualitative differences in the response to expansion with mutant peptide and are in line with the results from the killing assay presented earlier (Fig. 3L). In contrast, we found a set of genes including *GZMK, LTB, CD74*, *SELL*, *IFITM1*, *IFITM2* and *CX3CR1* expressed at higher levels in cells stimulated with mutant peptides (Fig S4F, S4H-S4K). Taken together, the scRNA-seq data indicate that stimulation with mutant peptide did not only lead to a reduced T cell response but also to altered gene expression patterns.

## DISCUSSION

The presented data demonstrate that SARS-CoV-2 may evade CTL surveillance through mutations in viral epitopes which lead to reduced peptide-MHC-I binding and quantitatively and qualitatively altered CTL responses. Deep SARS-CoV-2 genome sequencing results afford a valuable additional perspective that complements insights gained from numerous studies on SARS-CoV-2-specific T cell responses (*39*). Viruses employ numerous strategies to evade CD8^+^ T cell immune responses (*40*–*42*). The SARS-CoV-2 encoded ORF8 protein is hypothesized to down-regulate the surface expression of MHC-I molecules (*43*), and several reports linked mutations within the viral spike protein to the evasion of neutralizing antibody responses (*28*–*30*). Yet, we still lack a comprehensive understanding of the intrinsic capabilities of SARS-CoV-2 for immune evasion. Our study provides evidence that single nonsynonymous mutations in SARS-CoV-2 can subvert the immune response to CD8^+^ T cell epitopes. The majority of nonsynonymous mutations found in the validated CTL escapes had not reached fixation, i.e., were present at frequencies between 0.02 and 0.42 (Fig. 1B). This could be explained by the shorter duration of infection with SARS-CoV-2 compared to HIV or HCV. It may also reflect on the degree to which HLA polymorphism affects viral spreading within human populations. The impact of single anchor residue substitutions on the response of CD4^+^ T cells is still unclear.

Our findings do not rule out that substitutions of residues facing the cognate T cell receptor may give rise to the emergence of CTL neoepitopes. Of note, we could show for the YLQ epitope that T cell clones that expanded in vitro in the presence of mutant peptides were identical to those expanded in response to the wild type peptide, suggesting a similar if not identical structural basis underlying TCR-epitope engagement.

This study does not allow direct conclusions to be drawn concerning potential selection pressures which shape the mutational landscape of CD8^+^ T cell epitopes. This would invariably involve accounting for the HLA genotype of all individuals from whom SARS-CoV-2 genomes were sequenced. Moreover, how CTL escape mutations are maintained during transmission between individuals with differing HLA subtypes and how viruses carrying epitope mutations affect disease severity requires further investigation.

Many CTL epitopes for SARS-COV-2 have been described (*39*). Natural CTL responses against SARS-CoV-2 were associated with broad epitope recognition of on average 1.6 CD8^+^ T cell epitopes per antigen per HLA allele (*23*), which raises the question whether and how mutations in single epitopes affect virus control. This may be of particular importance for SARS-CoV-2 subunit vaccines, such as the RNA vaccines currently in use, which contain the S gene only and thus induce responses against a limited number of CD8 epitopes (*44*–*46*). In summary, our results highlight the capacity of SARS-CoV-2 to evade adaptive immune responses through sporadically emerging mutations in MHC-I epitopes.

## MATERIALS AND METHODS

### Study design

The objective of this study was to investigate mutations in SARS-CoV-2 for their potential to evade CD8^+^ T cell responses. For this study, we performed deep sequencing on virus samples from Austria. To characterize identified mutants, we performed in vitro MHC-I binding assays. Further, we performed functional assays on PBMCs isolated from COVID-19 patients. PBMCs were only analyzed from patients who were positive for HLA-A*02:01 or HLA-B*40:01. This study was performed in accordance with the recommendations of the Declaration of Helsinki. The protocols were approved by the Ethics Committee of the Medical University of Vienna, Austria (2283/2019 and 1339/2017) and written informed consent was obtained from all patients.

### Virus sample collection and processing

Virus samples were obtained from the Medical University of Vienna Institute of Virology, Medical University of Innsbruck Institute of Virology, Medical University of Innsbruck Department of Internal Medicine II and Division of Hygiene and Medical Microbiology, Central Institute for Medical-Chemical Laboratory Diagnostics Innsbruck, Klinikum Wels-Grieskirchen and the Austrian Agency for Health and Food Safety (AGES). Sample types included oropharyngeal swabs, nasopharyngeal swabs, tracheal secretion, bronchial secretion, serum and plasma. RNA was extracted using the following commercially available kits following the manufacturer's instructions: EasyMag (bioMérieux), MagMax (Thermo Fisher), MagNA Pure LC 2.0 (Roche), AltoStar Purification Kit 1.5 (Altona Diagnostics), MagNA Pure Compact (Roche) and QIAsymphony (Qiagen). Viral RNA was reverse-transcribed with Superscript IV Reverse Transcriptase (Thermo Fisher) and viral sequences were amplified with modified primer pools (*47*). PCR reactions were pooled and processed for high-throughput sequencing.

### PBMC sample collection and processing

Whole blood samples from hospitalized SARS-CoV-2-infected patients were collected at the Department of Medicine 4, Clinic Favoriten. Samples from the same individual were collected at 2- to 7-day time intervals to obtain sufficient blood volumes for different T cell analyses. Samples from healthy blood donors that were never exposed to SARS-CoV-2, were collected before the SARS-CoV-2 pandemic (June to November 2019). Peripheral blood mononuclear cells (PBMCs) were isolated by density gradient centrifugation and stored in liquid nitrogen until further use. HLA typing of PBMCs was carried out by next generation sequencing, as described previously (*48*).

### Virus sequencing, data processing and analysis

AMPure XP beads (Beckman Coulter) at a 1:1 ratio were used for amplicon clean-up. Amplicon concentrations and size distribution were assessed with the Qubit Fluorometric Quantitation system (Life Technologies) and the 2100 Bioanalyzer system (Agilent), respectively. After normalization of amplicon concentrations, sequencing libraries were generated with the NEBNext Ultra II DNA Library Prep Kit for Illumina (New England Biolabs) according to the manufacturer’s instructions. Library concentrations and size distribution were again assessed as indicated previously and pooled at equimolar ratios for sequencing. Sequencing was carried out on the NovaSeq 6000 platform (Illumina) on a SP flow cell with a read length of 2x250bp in paired-end mode.

After demultiplexing, FASTQ files were quality controlled using FASTQC (v. 0.11.8) (*49*). Adapter sequences were trimmed with BBDUK from the BBTools suite (https://jgi.doe.gov/data-and-tools/bbtools). Overlaps of paired reads were corrected with the BBMERGE from BBTools. Read pairs were mapped on the combined GRCh38 and SARS-CoV-2 genome (RefSeq: NC_045512.2) using BWA-MEM with a minimal seed length of 17 (v 0.7.17) (*50*). Only reads uniquely mapping to the SARS-CoV-2 genome were retained. Primer sequences were masked with iVar (*51*). The consensus FASTA file was generated from the binary alignment map (BAM) file using Samtools (v 1.9) (*52*), mpileup, Bcftools (v 1.9) (*52*), and SEQTK (https://github.com/lh3/seqtk). The read alignment file was realigned with the Viterbi method from LoFreq (v 2.1.2) for low-frequency variant calling (*53*). InDel qualities were added and low-frequency variants were called with LoFreq. Variants were filtered with LoFreq and Bcftools (v 1.9) (*54*). We only considered variants with a minimum coverage of 75 reads, a minimum phred-value of 90 and indels (insertions and deletions) with a HRUN of at least 4. Based on the control experiments described earlier, all analyses were performed on variants with a minimum alternative frequency of 0.02 (*55*). Variants were annotated with SnpEff (v 4.3) (*56*) and SnpSift (v 4.3) (*57*).

The output of LoFreq was filtered for nonsynonymous variants with a frequency cut-off of 0.02. The resulting mutations were then filtered for positions in reported CD8^+^ T cell epitopes. Data manipulation and plotting was carried out in R, with the packages dplyr, tidyr, ggplot2 and heatmap2.

### Identification of epitope mutations in SARS-CoV-2 genomes

Mutations in epitope regions were identified in all available protein alignment files for the SARS-CoV-2 proteins non-structural protein 3 (NSP3, n=164,819), NSP6 (n=164,806), M (n=164,846), spike (S, n=165,249), N (n=164,876) and E (n=164,847) retrieved on October 30, 2020, from the global initiative on sharing all influenza data (GISAID) database (*33*). Protein alignment files were first filtered for protein sequences that have less than 5% unknown amino acid positions. Epitope regions were then extracted from the alignment files and misaligned entries (>4 misaligned positions in epitope region) and protein sequences with more than 4 unknown positions in epitope regions were removed. Mutations in epitope regions were identified based on sequence comparison to the reference sequence “Wuhan-Hu-1” (GenBank: MN908947.3) (*58*).

### MHC-I binding predictions

To predict the binding strength of wild type and mutant peptides, NetMHCpan 4.1 was used (*32*). Briefly, wild type and mutant peptide sequences were interrogated for binding to HLA-A*02:01, HLA-A*02:06 and HLA-B*40:01 with the standard settings (strong binder % rank 0.5, weak binder % rank 2). The % ranks of wild type and mutant epitopes were then compared and plotted along the heatmap of variant frequencies.

### Peptides

Peptides were purchased from JPT Peptide Technologies GmbH or synthesized in-house, as indicated in Table S2. Peptides were produced in-house by solid-phase synthesis with the 9-fluorenyl-methoxy carbonyl (Fmoc)-method (CEM-Liberty and Applied Biosystems) on PEG-PS preloaded resins (Merck, Darmstadt, Germany) as previously described (*59*, *60*) with the following alterations. After synthesis the peptides were washed with 50 ml dichloromethane (Roth), cleaved from the resins using 28.5 ml trifluoroacetic acid (Roth), 0.75 ml silane (Sigma-Aldrich, St. Louis, MO, USA) and 0.575 ml H_2_O for 2.5 hours at room temperature (RT) and precipitated into pre-chilled *tert*-butylmethylether (Merck). The peptides were purified by reversed-phase high-performance liquid chromatography in a 10–70% acetonitrile gradient using a Jupiter 4 μm Proteo 90 Å LC column (Phenomenex) and an UltiMate 3000 Pump (Dionex) to a purity >90%. Their identities and molecular weights were verified by mass spectrometry (Microflex MALDI-TOF, Bruker).

### Synthesis of HLA/Peptide complexes.

cDNA encoding the extracellular domains of HLA-A*02:01 (UniProt: P01892) HLA-B*40:01 (UniProt: P01889) and beta-2-microglobulin (UniProt: P61769) were cloned without the leader sequence into pET-28b (HLA-A*02:01, HLA-B*40:01) and pHN1 (beta-2-microglobulin, β2m) for recombinant protein expression as inclusion bodies in *E. coli*. pET-28b was modified to encode a C-terminal 12x poly histidine tag (HIS_12_) or AviTag. Single colonies of *E. coli* (BL21) transformed with individual vectors were grown in 8l Luria-Bertani (TB) media at 37°C to an OD600 of 0.5. Protein expression was induced by addition of isopropyl β-D-thiogalactoside (IPTG, Sigma-Aldrich) to a final concentration of 1 μM. Cells were harvested after 4 hours of induction. Inclusion bodies containing HLA and β2m protein were isolated, fully denatured and refolded in vitro in the presence of ultraviolet light-cleavable peptides (UVCP; GILGFVFJL for HLA-A*02:01; TEADVQJWL for HLA-B*40:01; J= 3-amino-3-(2-nitro)phenyl-propionic acid) to produce HLA/UVCP protein (UVCP peptides: GILGFVFJL for HLA-A*02:01, TEADVQJWL for HLA-B*40:01, J= 3-amino-3-(2-nitro)phenyl-propionic acid) (*34*, *61*, *62*). The refolding reaction (500 ml) was dialyzed three times against 10 L of PBS. Dialyzed HLA/UVCP HIS_12_ tag proteins were purified by Ni^2+^-NTA agarose chromatography (HisTrap excel, GE Healthcare) followed by size exclusion chromatography (SEC) (Superdex 200 10/300 GL, GE Healthcare). Dialyzed HLA/UVCP AviTag proteins were concentrated to 2 ml using spin concentrators and purified by SEC. Purified HLA/UVCP AviTag proteins were biotinylated using biotin protein ligase BirA as described (*63*) and further purified by SEC. The purity and integrity of all proteins was confirmed via SDS-PAGE followed by silver staining.

### UV-mediated peptide exchange, Differential scanning fluorimetry (DSF) and tetramer synthesis

For peptide exchange, peptides were added to HLA/UVCP at an HLA/UVCP:peptide molar ratio of 1:20 (at a final concentration of 1.5 μM and 30 μM, respectively). For efficient cleavage, the reaction mix was placed within 5 cm from the CAMAG® UV Lamp 4 (Camag) and exposed to 366 nm UV light for 2h at 4°C followed by 16h incubation at 4°C.

For DSF, SYPRO Orange Protein Gel Stain (Thermo Fisher Scientific, 5000x stock solution) was diluted at 4°C into the solution containing UV-treated HLA/peptide HIS_12_ mixtures (see above) at a final concentration of 15x SYPRO Orange Protein Gel Stain. The reaction mix was immediately transferred to pre-chilled PCR tubes and placed on a CFX 96 Real-Time PCR system (BioRad) which had been precooled to 4°C. Samples were heated at a rate of 0.4°C/20 s and relative fluorescence units (rfu) were measured every 20s in the FRET channel. Readings were plotted as negative derivative of fluorescence change vs. temperature -d(RFU)/dT.

For tetramer synthesis fluorescence-labeled streptavidin was added to UV-exchanged HLA/peptide AviTag protein solution in 10 steps as published (*64*).

### Flow cytometry assays following 10-12d in vitro stimulation

For in vitro expansion, cryopreserved PBMCs were thawed in pre-warmed RPMI-1640 medium (R0883, Sigma) containing 10% FBS (FBS 12-A, Capricorn), 10 mM Hepes (Sigma), 2 mM GlutaMAX (Gibco), 50 IU/ml Pen-Strep (Sigma) and 50 IU IL-2 (Peprotech) at a concentration of 1×10^6^ cells/ml. PBMCs were pulsed with peptides (1 μg/ml) and cultured for 10-12 days adding 100 IU IL-2 on day 5. In vitro expanded cells were analyzed by intracellular cytokine and cell surface marker staining. PBMCs were incubated with 2 μg/ml of peptide and 1 μg/ml anti-CD28/49d antibodies (L293 and L25, Becton Dickinson) or with CD28/49d antibodies alone (negative control) for 6h. After 2h, 0.01 μg/ ml brefeldin A (Sigma) was added. Staining was performed using APC/H7 anti-human CD3 (SK7, Becton Dickinson), Pacific Blue anti-human CD4 (RPA-T4, Becton Dickinson), PE anti-human CD8 (HIT8a, Becton Dickinson), FITC anti-human IFN-γ monoclonal antibodies (25723.11, Becton Dickinson), and Fix/Perm kit (Invitrogen). Viable cells were determined using live/dead cell viability assay kit (Invitrogen). Tetramer staining (10 μg/ml) was performed for 60 min at 4°C followed by staining with anti-CD8α antibody (OKT8, Invitrogen) (10 μg/ml) for 30 min at 4°C.

Flow-cytometric analyses were carried out on Cytek® Aurora (Cytek® Biosciences) or FACS Canto II (Becton Dickinson) instruments and evaluated using FlowJo software v. 7.2.5 (Tree Star). The gate for detection of IFN-γ in peptide-stimulated cell samples was set in the samples with costimulation only.

### IFN-γ ELISpot assay

For ex vivo ELISpot assays, PBMCs were thawed and depleted of CD4^+^ cells using magnetic microbeads coupled to anti-CD4 antibody and LD columns according to the manufacturer´s instructions (Miltenyi Biotec). A total of 1-2 × 10^5^ CD4-depleted cells per well were incubated with 2 μg/ml single peptides, AIM-V medium (negative control) or PHA (L4144, Sigma; 0,5 μg/ml; positive control) in 96-well plates coated with 1.5 μg anti-IFN-γ (1-D1K, Mabtech). After 45h incubation, spots were developed with 0.1 μg biotin-conjugated anti-IFN-γ (7-B6-1, Mabtech), streptavidin-coupled alkaline phosphatase (Mabtech, 1:1000), and 5-bromo-4-chloro-3-indolyl phosphate/nitro blue tetrazolium (Sigma). Spots were counted in 2-3 wells per sample using a Bio-Sys Bioreader 5000 Pro-S/BR177 and Bioreader software generation 10. T cell responses were considered positive when mean spot counts were at least threefold higher than the mean spot counts of three unstimulated wells.

### Generation of autologous EBV-transformed lymphoblastoid B cell lines

PBMCs were isolated from heparinized blood of four COVID-19 convalescent patients (SARS048, SARS047, SARS044, SARS050) by standard Ficoll density gradient centrifugation using Lymphoprep (Technoclone). EBV-transformed lymphoblastoid B cell lines (EBV^+^ B cells) were generated by supplementing PBMCs with infectious marmoset P95-8 supernatant (ATCC) plus 200 ng/ml cyclosporin A (Sandimmune) in the presence of ODN2006 (1 μg/ml; InvivoGen) at 37°C in 5% CO_2_. After 10 days of incubation, cells were replated 1:2 in fresh medium and further cultured in RPMI 1640 medium supplemented with 10% fetal calf serum, 2 mM *L*-glutamine and 100 μg/ml gentamicin sulfate.

### Cellular cytotoxicity assay

The cytolytic activity of CTLs was tested in standard ^51^Cr-release assays. Autologous EBV^+^ B cell cells (2 × 10^6^/2 ml, 24-well) were pulsed with YLQ wild type (YLQPRTFLL) or mutant peptide (YFQPRTFLL) or a negative control peptide (GVIMMFLSLGVGA, a non-immunogenic yellow fever virus peptide), respectively, at a concentration of 1μg/ml overnight. On the next day, cells were harvested, resuspended in 100 μl medium and labeled with 150 μCi of Na^51^CrO^4^ (PerkinElmer) at 37°C for 3 hours. After four washes, autologous EBV^+^ B cells were added to round bottom 96-well plates that contained titrated numbers of wild type or mutant YLQ peptide specific PBMCs, generated as described under “Flow cytometry assays following 10-12d in vitro stimulation” (in duplicates). Subsequently, plates were centrifuged at 200 g for 5 min. After 5 hours of incubation at 37°C, the supernatants were collected, and the radioactivity was determined in a γ-counter (Packard). The percentage of specific release was determined as follows: [CTL-induced release (cpm) − spontaneous release (cpm)]/[maximum release (cpm) − spontaneous release (cpm)] × 100.

### Tetramer sorting, Hashtag labeling and single-cell RNA sequencing

PBMCs were harvested after 10-12d of in vitro expansion (as described under “Flow cytometry assays following 10-12d in vitro stimulation”) with either YLQ wild type (YLQPRTFLL) or mutant peptide (YFQPRTFLL) and counted using TruCount tubes (Becton Dickinson) on a FACS Fortessa (Becton Dickinson). Cells were centrifuged at 400xg for 5 min at 4°C and washed in ice-cold FACS buffer (DPBS (Gibco) containing 1% Octaplas® LG, blood group AB (Octapharma). All staining and washing steps were performed on ice. After centrifugation, the cell pellet was resuspended in 50 μl FACS buffer and stained with HLA-A*02:01 YLQ wt (YLQPRTFLL) tetramer in PE at 10 μg/ml for 20 min. Cells were washed with ice-cold FACS buffer followed by staining with FITC anti-human CD8 (SK1, Becton Dickinson) for 20 min. Cells were washed with ice-cold FACS buffer, resuspended in 200 μl FACS buffer and dead cells were counterstained with 2 μg/ml 4’,6-diamidin-2-phenylindol (DAPI) (Sigma-Aldrich). Cell sorting was performed on a FACS Aria II Cell Sorter (Becton Dickinson). For each patient tetramer positive and tetramer negative cells of both wt peptide expanded and mutant peptide expanded cultures were sorted into 50 μl sorting buffer (DPBS (Gibco) with 0,08% bovine serum albumin (BSA) (Sigma-Aldrich). Cells were centrifuged and the supernatant was carefully discarded leaving 50 μl behind. To this residual 50 μl, 1 μl (0.5 μg) TotalSeq-C0251 anti-human Hashtag Antibody 1 to 4, respectively, (394661, 394663, 394665, 394667, all BioLegend) was added and incubated for 20 min on ice. Cells were washed in 1 ml ice-cold sorting buffer and centrifuged and taken up in 1 ml sorting buffer. Cells were counted again using TruCount tubes.

For each patient volumes corresponding to 22,000 cells of each condition were pooled and centrifuged again. The cell pellet was resuspended in 80 μl sorting buffer and stored on ice until further preparation for sequencing.

Single-cell RNA-seq was performed on the live samples using the 10x Genomics Chromium Single Cell Controller with the Chromium Single Cell 5′ v1.1 kit following the manufacturer’s instructions. After cDNA amplification TCR enrichment and enrichment of feature barcoding sequences from the hashtag-antibodies were performed according to instructions by 10X Genomics manufacturer’s guidelines for VDJ and feature barcoding enrichment. After quality control, libraries were sequenced on the Illumina NovaSeq sequencing platform using the SP flow cell in 2x150bp paired-end mode at the Biomedical Sequencing Facility (BSF) of the CeMM Research Center for Molecular Medicine of the Austrian Academy of Science.

Primary analysis was done using the CellRanger v5.0.1 software (10X Genomics). Alignment to the human reference transcriptome (refdata-gex-GRCh38-2020-A for gene expression and vdj_GRCh38_alts_ensembl-5.0.0 for VDJ analysis) was performed by the BSF. Hashtag Oligo identification was performed with the CITE-seq software (*65*) and demultiplexing was done with custom Python scripts by the BSF. We used the R statistics software to perform all further analysis using the Seurat package version 3.9.9.9038 (*66*). Briefly, CellRanger outputs from both patients were jointly loaded into R to perform quality control (removing cells with less than 1,000 genes, mitochondrial content more than 10%, as well as all cells without [negatives] or with two conflicting hashtag labels [doublets]). Preliminary clustering revealed two outlier clusters of cells shared across all conditions with higher number of counts (Fig. S5) which dominated the variance and were removed from downstream analysis. Patient-dependent batch effects were then removed by integration with Seurat’s FindIntegrationAnchors and Integrate data (nfeatures=2000, dims=1:30). The dataset was then normalized (function NormalizeData, ScaleData) to generate corrected, log-transformed relative cell counts. Integrated data was used for low-dimensional projection using UMAP (*67*) based on the top 10 principal components and for clustering cells (resolution = 0.6). Differential gene expression analysis was performed using the FindMarkers function (method: MAST). The adjusted p-values returned by FindMarkers were further subjected to Bonferroni correction for the two tests applied (YLQ+ vs. YLQ- and WT YLQ+ vs. MUT YLQ+). For plotting, P-values smaller than 10^−350^ were capped to 10^−350^. The results of the differential expression analysis on raw counts (|log2 fold-change| > 0.25) are reported in Table S7. Gene signatures for cytotoxic, viral, unhelped, IFN-γ and exhaustion responses were obtained from Kusnadi *et al*. (*38*), and enrichment was evaluated using R package AUCell (*68*).

For T cell clonotype analysis, the results from CellRanger were loaded into R and processed using the scRepertoire package and custom code. Clonotype identity was determined by the amino acid sequence of the assembled receptor sequences and we focused our analysis on the five most frequent clonotypes for each of the two individuals (Table S8).

### Statistical Analysis

Statistical analysis of differences between the wild type and mutant CD8^+^ T cell responses for ELISpot and ICS was done with Wilcoxon matched-pairs signed rank test. For comparison of >1 mutant responses, a Generalized Equation Estimations (GEE) model with peptide (fixed factor) and patient (random factor) was used. For analysis of CTL killing assays 2-way ANOVA followed by Dunnett’s multiple comparison test was used. Differential gene expression analysis was performed using the FindMarkers function (method: MAST). The adjusted p-values returned by FindMarkers were further subjected to Bonferroni correction for the two tests applied (YLQ+ vs. YLQ- and WT YLQ+ vs. MUT YLQ+).

## Supplementary Materials

immunology.sciencemag.org/cgi/content/full/6/57/eabg6461/DC1 Figure S1. Supplementary figures for mutation analysis Figure S2. Controls and additional DSF assay results Figure S3. Supplementary figures for PBMC analysis Figure S4. Supplementary figures for scRNA-seq analysis Figure S5. Quality control plots for scRNA-seq analysis and outlier clusters Table S1. Wild type epitopes investigated in this study Table S2. Samples with epitope mutations at allele frequency >0.02 (Excel spreadsheet) Table S3. Peptides used in the study and their % rank predicted by netMHCpan v4.1 Table S4. T_m_ values for peptides tested in DSF assay Table S5. Characteristics of HLA-A*02:01/HLA-B*40:01 positive patients Table S6. HLA genotypes Table S7. Overview of ELISpot results for wild type peptides Table S8. DEGs from scRNA-seq analysis (Excel spreadsheet) Table S9. Top clonotypes from TCR sequencing (Excel spreadsheet) Table S10. Acknowledgments for sequences downloaded from GISAID (separate PDF file) Table S11. Raw data (Excel spreadsheet) Reproducibility checklist
